# A pharmacometric pulmonary model predicting the extent and rate of distribution from plasma to epithelial lining fluid and alveolar cells—using rifampicin as an example

**DOI:** 10.1007/s00228-014-1798-3

**Published:** 2015-01-27

**Authors:** Oskar Clewe, Sylvain Goutelle, John E. Conte, Ulrika S. H. Simonsson

**Affiliations:** 1 Department of Pharmaceutical Biosciences, Uppsala University, PO Box 591, Uppsala, 75124 Sweden; 2 Hospices Civils de Lyon, Groupement Hospitalier de Gériatrie, Service Pharmaceutique—ADCAPT, Francheville, France; 3 Université Lyon 1, CNRS, UMR5558, Laboratoire de Biométrie et Biologie Evolutive, F-69622 Villeurbanne, France; 4 Department of Epidemiology & Biostatistics, University of California at San Francisco, San Francisco, CA USA; 5 American Health Sciences, San Francisco, CA USA; 6 Université Lyon 1, ISPB–Faculte´ de Pharmacie , Lyon, France

**Keywords:** Pharmacometrics, Pulmonary distribution, Bronchoalveolar lavage, Epithelial lining fluid, Alveolar cells

## Abstract

**Purpose:**

The purpose of the study was to develop a drug-unspecific approach to pharmacometric modeling for predicting the rate and extent of distribution from plasma to epithelial lining fluid (ELF) and alveolar cells (AC) for data emanating from studies involving bronchoalveolar lavage (BAL) sampling, using rifampicin (RIF) as an example.

**Methods:**

Data consisting of RIF plasma concentrations sampled at approximately 2 and 4 h postdose and ELF and AC concentrations quantified from one BAL sample, taken at approximately 4 h postdose, in 40 adult subjects without tuberculosis was used as an example for model development.

**Results:**

This study emphasized the usage of drug-specific plasma pharmacokinetics (PK) for a correct characterization of plasma to pulmonary distribution. As such, RIF PK was described using absorption transit compartments and a one compartment distribution model coupled with an enzyme turn-over model. The ELF and AC distribution model consisted of characterization of the rate of distribution of drug from plasma to ELF and AC by two distribution rate constant, *k*_ELF_ and *k*_AC_, respectively. The extent of distribution to ELF and AC was described by unbound ELF/plasma concentration ratio (*R*_ELF/unbound-plasma_) and unbound AC/plasma concentration ratio (*R*_AC/unbound-plasma_) which typical values were predicted to be 1.28 and 5.5, respectively.

**Conclusions:**

The model together with a drug-specific plasma PK description provides a tool for handling data from both single and multiple BAL sampling designs and directly predicts the rate and extent of distribution from plasma to ELF and AC. The model can be further used to investigate design aspects of optimized BAL studies.

## Introduction

Pulmonary distribution is of high interest in the field of antituberculosis drugs. Even though tuberculosis is not a lung-exclusive disease, and can affect various organs and tissues, pulmonary tuberculosis is the most common and perhaps most recognized form of the disease. Many of the first-line drugs today used in treatment of pulmonary tuberculosis were launched in the 1950s and 1960s, and naturally, little was then known about the rate and extent of pulmonary penetration and the correlation between plasma concentrations and concentrations at the site of action. Today tuberculosis is still a major and global health problem, ranked as the second leading cause of death from an infectious disease worldwide [1]. The development of new antibiotics is not matching the incline in resistance against the available antibiotics today. To illustrate, the approval of bedaquiline by the FDA marked the first new antitubercular agent approved in over 40 years and this for a disease that in 2012 alone was estimated to have been developed in 8.6 million people worldwide [1].

An example of a problem potentially emanating from the lack of integration of pharmacokinetic (PK) and pharmacodynamic (PD) analysis in drug development for drugs launched in the 1950s and 1960s is the today widely recognized suboptimal dosing of rifampicin (RIF) [2, 3]. RIF is one of four drugs that make up the current first-line antituberculosis drug therapy for treatment-naïve adults recommended by the world health organization (WHO). The PK of RIF is well characterized in plasma [4, 5] and with the possibility brought by new methods and techniques, there today exists a number of publications reporting drug concentrations quantified from samples taken from the pulmonary tract both for RIF [6, 7] and review articles for antimicrobials in general [8, 9]. Despite this, for many drugs, the effects of active transporters, disease-afflicted tissue, and the differences in the efferent of sampling and quantification methods are still to some extent uncharacterized and not well understood. Shedding new and more light on these still unclear areas is an important part of the research focused on finding and describing the important relationship between PK and PD, such as clinical and microbiological outcomes.

Bronchoalveolar lavage (BAL) is used in both drug research and in clinical practice as a way to quantify drug concentrations from epithelial lining fluid (ELF) [6, 10] and alveolar cells (AC) [6, 11]. Information about the extent of distribution, often parameterized as ELF/plasma and AC/plasma concentration ratios, is of great value in order to assess whether sufficient drug concentrations are achieved at extracellular (ELF) and intracellular (AC) sites. The volumes of cells in ELF has been reported to consist of 83 % macrophages, 17 % lymphocytes, and 1 % neutrophils [9] making quantified AC concentrations mainly reflect the concentrations in macrophages. However, it is not only the extent of distribution to the lung that is of importance. Capturing the rate of distribution from plasma to the lung is important when obtaining relationships between PK and PD. This is especially relevant for drugs and compounds without an instantaneous or fast equilibrium between plasma and the lung, where the exposure in plasma may not be a good marker of the drug exposure at the site of action and could potentially distort PKPD relationships.

Obviously, there is a need for more innovative approaches and high quality research targeting both existing treatments as well as novel antitubercular drugs. The aim of the present work was hence to develop a general pharmacometric pulmonary model for predicting the extent and rate of drug distribution from plasma to ELF and AC using RIF as an example. Such a model will have the potential to increase knowledge about distribution and PKPD properties of existing drugs as well as provide a useful tool in development of novel compounds targeting tuberculosis.

## Material and methods

### Data

Data from a previously published study [6] were used in this pharmacometric analysis. Written informed consent, according to the guidelines of the Institutional Review Board of The University of California, San Francisco, USA, was obtained. The study consisted of 40 adult subjects without tuberculosis and included 10 women without acquired immunodeficiency syndrome (AIDS), 10 men without AIDS, 10 women with AIDS, and 10 men with AIDS. The subjects received 600 mg RIF orally once a day for 5 days. Rifampicin plasma concentrations were measured on day 5 at approximately 2 and 4 h postdose. Rifampicin concentrations in ELF and AC recovered by BAL were measured at approximately 4 h after administration of the last dose. Rifampicin concentrations were measured by high-performance liquid chromatography [12]. For the determination of the RIF concentrations in ELF and AC, the volume of the ELF recovered by BAL was calculated by using the urea dilution method [13], while the volume of AC was estimated from the cell count performed in BAL fluid [6].

### Pharmacokinetic modeling and model evaluation

Data analysis was performed using the software NONMEM, version 7.2 (ICON Development Solutions, Ellicott City, USA) [14], using the first order conditional estimation method with interaction (FOCE INTER). R (version 3.0.1) [15] was used for graphical analysis and data management. PsN 3.6.9 [16–18] was used for prediction-corrected visual predictive checks (pcVPC). Xpose (version 4.4.1) [19] was used for visualization of data and results. Numerical model comparison and a run record was utilized and maintained with the software Pirana (version 2.7) [20].

A total of 76 plasma, 32 ELF, and 36 AC concentrations were included in the analysis. The model building process was performed in a stepwise fashion, starting from the previously published RIF PK enzyme turn-over model in order to account for RIF autoinduction [5]. The model consisted of a one compartment disposition model with absorption transit compartments. The mean transit time (MTT) and the number of transit compartments (N) were fixed to previously published values [5] due to insufficient sampling in the absorption phase. RIF plasma concentrations increased the enzyme production rate (*k*_ENZ_), which in turn increased the enzyme pool in a non-linear fashion by means of an *E*_MAX_-model. The parameters related to the autoinduction of RIF oral clearance (*CL/F*) were also fixed to the previously published values [5], as the data contained only 5 days of treatment, and as such only limited information about the time to steady-state (approximately 40 days) [5] was available. The impact of allometric scaling was assessed by using four different basic turn-over models: (1) no scaling, (2) allometric scaling using bodyweight as the size descriptor, (3) allometric scaling using normal fat mass (NFM) as the size descriptor, and (4) allometric scaling using fat free mass (FFM) as the size descriptor. The typical value (TV) of *CL/F* and apparent central volume of distribution (*Vc/F*) were scaled allometrically standardized to each of the size descriptors using Eqs. 1 and 2, respectively:

1 $$ \mathrm{T}\mathrm{V}\left(\frac{\mathrm{CL}}{F}\right)={\left(\frac{\mathrm{CL}}{F}\right)}_{\mathrm{STD}}\times {\left(\frac{{\mathrm{MASS}}_{\mathrm{i}}}{70}\right)}^{\frac{3}{4}} $$

2 $$ \mathrm{T}\mathrm{V}\left(\frac{V_C}{F}\right)={\left(\frac{V_C}{F}\right)}_{\mathrm{STD}}\times {\left(\frac{{\mathrm{MASS}}_{\mathrm{i}}}{70}\right)}^1 $$

where MASS_i_ denotes individual values of the three size descriptors, bodyweight, NFM, and FFM used in the respective basic models. (*CL/F*)_STD_ is the typical oral clearance at pre-induced state in a patient weighing 70 kg and (*V*c/F)_STD_ is the typical volume of distribution in a patient weighing 70 kg.

Individual FFM values (FFM_i_) were calculated as:

3 $$ {\mathrm{FFM}}_{\mathrm{i}} = \frac{{\mathrm{WHS}}_{\mathrm{MAX}}\times {\mathrm{HT}}^2\times \mathrm{W}\mathrm{T}}{{\mathrm{WHS}}_{50}\times {\mathrm{HT}}^2+\mathrm{W}\mathrm{T}} $$

where the maximal weight height squared (WHS_MAX_) is 42.92 kg/m^2^ and WHS_50_ is 30.93 kg/m^2^ in men. WHS_MAX_ is 37.99 kg/m^2^ and WHS_50_ is 35.98 kg/m^2^ in women. HT is height in meters. NFM was expressed differently for *CL/F* and *V/F* as described by Andersson and Holford [21]:

4 $$ {\mathrm{NFM}}_{\mathrm{i}}={\mathrm{F}\mathrm{FM}}_{\mathrm{i}}+{\left(\mathrm{Ffat}\right)}_{\frac{\mathrm{CL}}{\mathrm{F}}}\times \left({\mathrm{WT}}_{\mathrm{i}}-{\mathrm{F}\mathrm{FM}}_{\mathrm{i}}\right) $$

5 $$ {\mathrm{NFM}}_{\mathrm{i}}={\mathrm{FFM}}_{\mathrm{i}}+{\left(\mathrm{Ffat}\right)}_{\frac{V}{F}}\times \left({\mathrm{WT}}_{\mathrm{i}}-{\mathrm{FFM}}_{\mathrm{i}}\right) $$

where ${\left(\mathrm{Ffat}\right)}_{\frac{\mathrm{CL}}{\mathrm{F}}}$ and ${\left(\mathrm{Ffat}\right)}_{\frac{\mathrm{V}}{\mathrm{F}}}$ denote the estimated contribution of fat mass to $\frac{\mathrm{CL}}{\mathrm{F}}$ and $\frac{\mathrm{V}}{\mathrm{F}}$, respectively.

The ELF and AC drug penetration was described using effect compartments [22]:

6 $$ \frac{d{C}_{\mathrm{ELF}}}{dt} = {k}_{\mathrm{ELF}}\times \left({R}_{\mathrm{ELF}/\mathrm{plasma}}\times \frac{A_{\mathrm{plasma}}}{V_{\mathrm{plasma}}}-{C}_{\mathrm{ELF}}\right) $$

7 $$ \frac{d{C}_{\mathrm{AC}}}{dt} = {k}_{\mathrm{AC}}\times \left({R}_{\mathrm{AC}/\mathrm{plasma}}\times \frac{A_{\mathrm{plasma}}}{V_{\mathrm{plasma}}}-{C}_{\mathrm{AC}}\right) $$

where *C* is concentration, *k*_ELF_ and *k*_AC_ are the rate constants for the transfer of drug from the plasma to ELF or AC, respectively. *R*_ELF/plasma_ and *R*_AC/plasma_ are the ELF/plasma and AC/plasma concentration ratios, respectively, at pseudo steady-state. *A*_plasma_ */V*_plasma_ is the concentration of drug predicted in the plasma compartment at time *t*, with *A*_plasma_ being the amount of drug in plasma and *V*_plasma_ being the apparent plasma volume of distribution. To derive the ratio of ELF and AC to unbound plasma concentrations, (*R*_ELF/unbound-plasma_) and (*R*_AC/unbound-plasma_), respectively, *R*_ELF/plasma_, and *R*_AC/plasma_ were divided by free fraction of RIF in plasma (0.2) [23]. The protein concentration in ELF has previously been reported to be between 3.9 and 8 mg/ml, depending on disease state, representing only 6–12 % of the plasma concentration for children with and without congestive heart disorder [24]. In a similar study with healthy infants, similar results were reported with regards to protein concentration in ELF, 3.3–4.6 mg/ml depending on sampling technique [25]. The exact protein binding will naturally vary with different populations but based on the low concentration reported for children and lacking a more relevant value for this population, the protein binding in ELF and AC was judged to be negligible and hence not taken into account.

The likelihood ratio test (LRT) was used to evaluate statistical significance for inclusion of additional parameters in nested models, where the objective function value (OFV) is assumed to be *χ* ^2^ distributed. A decrease in the OFV of 3.84 points between hierarchical models with one parameter differing was considered as a statistical difference at the 5 % significance level. Model selection was guided by goodness-of-fit plots, pcVPCs, parameter precision, and scientific plausibility.

## Results

**Fig. 1:**
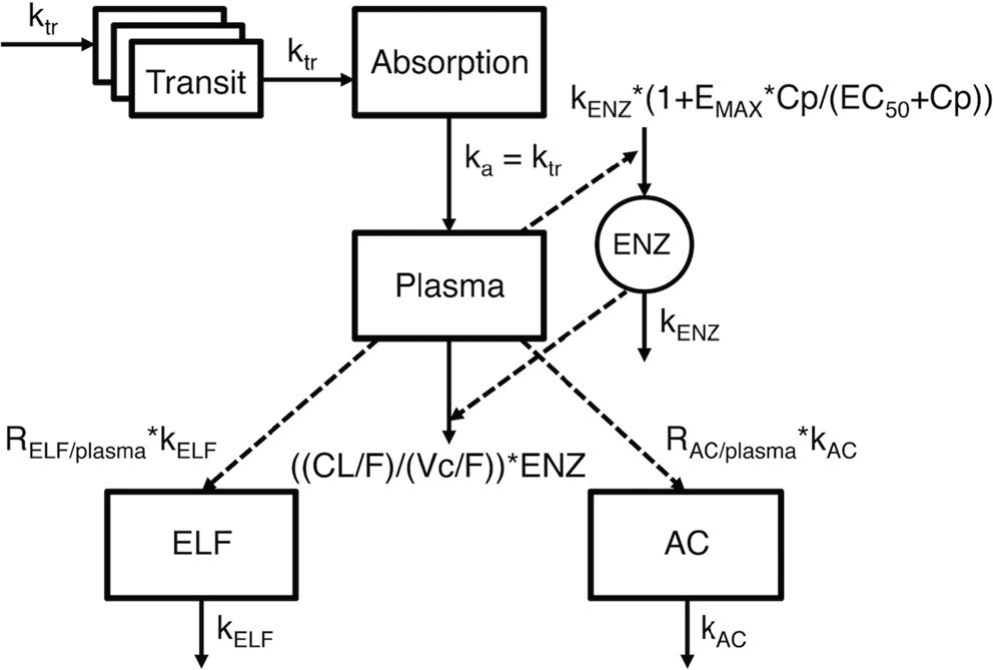
Schematic representation of the final rifampicin (RIF) lung and plasma pharmacometric submodels. Drug is transferred via a number of transit absorption compartments to the absorption compartment and further via the rate constant *k*_a_ to the central plasma compartment. Rifampicin autoinduction was modeled with an enzyme turn-over model in which the RIF plasma concentrations increased the enzyme production rate (*k*_*ENZ*_) which in turn increased the enzyme pool in a non-linear fashion by means of an *E*_MAX_-model. Cp is the RIF plasma concentration and *E*_*max*_ is the maximal autodinduction of *CL/F*. *EC*_*50*_ is the RIF concentration resulting in 50 % of the maximal autoinduction of *CL/F*. The ELF and AC drug penetration sub models were described using two parameters for each submodel. The rate of distribution of drug from plasma to ELF and AC was captured by two distribution rate constant, *k*_ELF_ and *k*_AC_, respectively. The extent of distribution to ELF and AC was described by unbound ELF/plasma concentration ratio (*R*_ELF/unbound-plasma_) and unbound AC/plasma concentration ratio (*R*_AC/unbound-plasma_)

A schematic representation of the final pharmacometric pulmonary model is shown in Fig. 1.

The final model included allometric scaling using FFM. This model had 22 points lower OFV compared to a model without scaling. Models with allometric scaling using NFM and bodyweight had an OFV drop of 22 and 18, respectively, compared to the model without scaling. Scaling with FFM and NFM had the same OFV value. However, the former was selected due to fewer parameters compared to a model with NFM scaling.

To mimic an almost instantaneous distribution from plasma to the lungs, the rate constants for the transfer of drug from the plasma to ELF or AC, *k*_ELF_ and *k*_AC_, respectively, were fixed to an equivalent of an equilibration half-life of about 1 min (instantaneous). This was needed as the sample design included only one sample per subject that was taken approximately at the same time (4 h) in all subjects wherefore the rate of distribution could not be estimated. The sampling time point of 4 h was also judged to occur when distribution equilibrium had been reached between plasma, ELF, and AC. Since the lung is a highly perfused organ and that this assumption of the rate of distribution does not affect the estimate of the extent of distribution, the assumption is valid for estimation of the extent of distribution at 4 h.

**Fig. 2:**
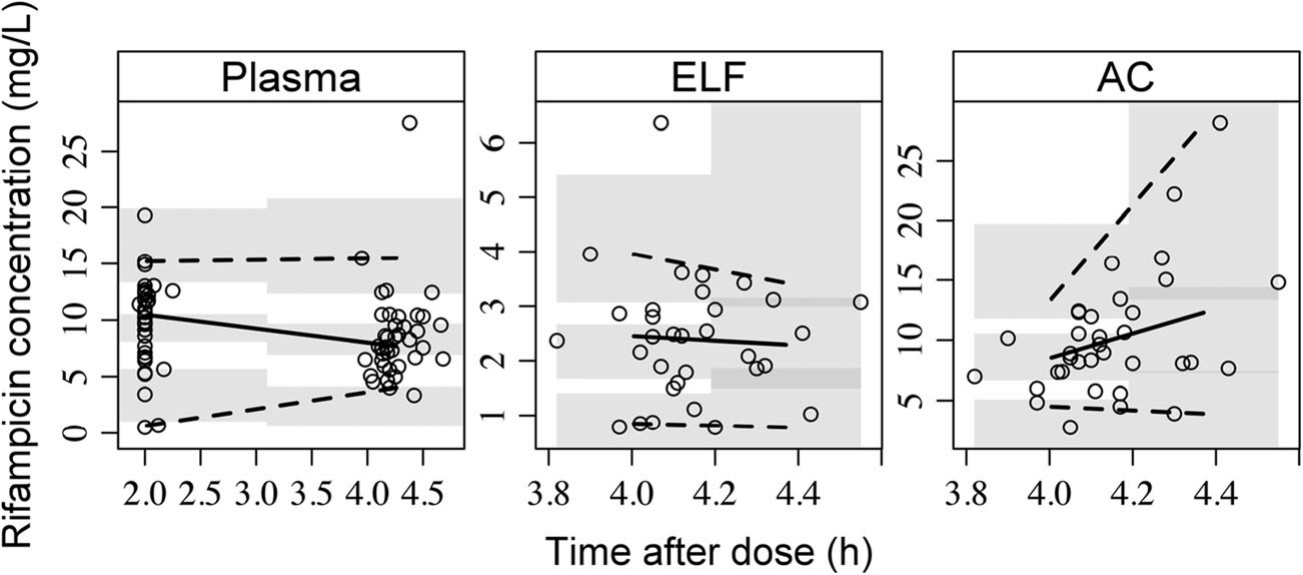
Prediction-corrected visual predictive check of the final RIF plasma, ELF and AC pharmacometric submodels. The *open circles* are the population prediction-corrected observations. The *solid line* is the median of the observed data and the dashed lines are the 5th and 95th percentiles of the observed data. The *top and bottom shaded areas* are the 95 % confidence intervals for the 5th percentile and the 95th percentile of simulated data. The *middle shaded area* is the 95 % confidence interval for the median of the simulated data

The final model predicted the plasma, AC, and ELF data well (Fig. 2). The parameter estimates of the final model are shown in Table 1. The *R*_ELF/plasma_ and *R*_AC/plasma_ were predicted to be 0.26 and 1.1, respectively. When adjusting for free fraction of 20 % in plasma [23], the unbound *R*_ELF/unbound-plasma_ and *R*_AC/unbound-plasma_ ratios were predicted to be 1.28 and 5.5, respectively. Interestingly, when the *R*_ELF/plasma_ and *R*_AC/plasma_ were not corrected for unbound fraction in plasma, the model predicted the exposure in ELF to be lower than in plasma whereas the reverse was seen when accounting for the protein binding in plasma.

**Table 1:** Parameter estimates and relative standard errors of the final model

Parameter	Estimate (95 % CI)	Relative standard error (%)
TV(*CL/F*)_STD_ (L h^−1^)	3.85 (2.26–8.68)	3.1
TV(*Vc/F*)_STD_ (L)	76.6 (60.85–88.83)	2.7
MTT (h)	0.71^a^	
*N*	1^a^	
*E*_MAX_	1.04^a^	
EC_50_ (mgL^−1^)	0.0705^a^	
*k*_ENZ_ (h^−1^)	0.0036^a^	
*k*_ELF_ (h^−1^)	41.58^a^	
*R*_ELF/plasma_	0.26 (0.21–0.31)	4.3
*R*_ELF/unbound-plasma_	1.28^b^	
*k*_AC_ (h^−1^)	41.58^a^	
*R*_AC/plasma_	1.1 (0.92–1.35)	6.2
*R*_AC/unbound-plasma_	5.5^c^	
IIV_CL/F_ (%)	88.8 (9.43–106.77)	24.2
Plasma proportional error (%)	35.2 (25.11–45.42)	3.6
ELF proportional error (%)	40.7 (30.26–54.76)	2.9
AC proportional error (%)	37.1 (22.95–46.91)	7.3

*IIV* interindividual variability expressed as coefficient of variation, *RSE* relative standard error reported on the approximate standard deviation scale

^a^Fixed parameter

^b^Calculated post estimation as *R*_ELF/plasma_ divided by the free fraction in plasma (20 %) [23]

^c^Calculated post estimation as *R*_AC/plasma_ divided by the free fraction in plasma (20 %) [23]

## Discussion

The semi-invasive nature of the BAL technique makes repeated sampling from the same subject more problematic than compared to repeated sampling using a non-invasive method. This leads to that many studies involving BAL sampling has only one or a few samples per subject available to quantify the distribution of the drug from plasma to ELF and AC. This poses a problem as a traditional pharmacokinetic compartment model approach for plasma as well as lung observation often involves describing the distribution of drug over time. When as in this case a one sample per subject design have been utilized, the traditional compartmental approach, unless all subjects are sampled at very different time points, will not be able to quantify the compartmental model parameters with a high accuracy or precision. Well aware of this limitation and potential lack of available tools to handle such data, we have developed a general pharmacometric pulmonary model that, together with a substance-specific plasma PK model, can be used with both one sample per subject as well as more rich BAL sampling designs focusing on characterizing the concentration ratios between ELF/plasma and AC/plasma instead of the distribution of drug over time. The concentration ratios are measurement of the extent of distribution which is, in this pharmacometric pulmonary model, directly obtained with parameter precision. In addition, the rate of distribution can be quantified using an optimized sampling design or fixed to instantaneous distribution if samples are only taken as in this design at one late time point resembling a pseudo steady-state. In such situation, only the extent of distribution will be quantified which was the situation in this example where rifampicin were sampled in ELF and AC at 4 h postdose. In this analysis example, no IIV was quantified for the parameters describing the rate or extent of distribution. In order to allow for IIV to be quantified with good precision, more than one sample per subject is needed.

Our suggested approach is not drug specific and can be applied to any drug as long as the plasma PK submodel is optimized for the particular drug. When assessing the distribution of drug from as in this case plasma to pulmonary tissue, it is not only important to in a correct way describe the pulmonary distribution but also strive to as correctly as possible describe the plasma PK properties of the drug. Failure of capturing the plasma PK properties of the drug will not only bias the description of the plasma concentrations of the drug but also the relationship of drug concentrations in plasma and the pulmonary tract. It is thus equally important to develop an as true as possible plasma PK submodel as the submodels relating to the pulmonary concentrations. The plasma PK submodel in this work was based on a previously published RIF pharmacometric model that included auto induction [5]. Without inclusion of the enzyme component in the plasma PK submodel that accounts for the autoinduction, the description of the plasma to ELF and AC concentration ratios would have been biased. As with all analysis involving the use of previously published approaches, estimations using the new study population where performed where possible. As the data used in this analysis were sparse and only included two plasma samples per subject, estimation of all parameters were not possible. These parameters where then fixed to the values reported by the authors of the original publication [5]. Evaluation of allometric scaling of *CL/F* and *V/F* with bodyweight, FFM, and NFM resulted in similar OFVs for the models using NFM and the FFM size descriptor and a higher OFV value using scaling with bodyweight. The FFM scaling method was judged superior based on the two extra parameters required by the NFM method.

The dataset used for model development included 40 subjects with ELF and AC concentrations quantified from one BAL sample per subject. In addition to the sparseness of the data, the difference in interindividual sampling times was very small. In the estimation of the unbound concentration ratios *R*_ELF/plasma_ and *R*_AC/plasma_, two assumptions regarding protein binding were made. The concentration ratios were adjusted for protein binding based on that only the free fraction of a drug is able to distribute from blood to the interstitial fluid and that this unbound drug concentration is believed to be more closely associated to drug efficacy. In the model, no adjustment was made for protein binding in ELF or AC. The protein levels in ELF have previously been shown to be low and even though potentially present, the effect from it was judged to be negligible, wherefore most likely the total and the unbound concentrations of drugs in ELF are similar [9]. No information was available regarding the protein binding in AC, and it was therefore assumed to be negligible as in ELF. The parameters *R*_ELF/plasma_ and *R*_AC/plasma_ in the model represent the concentration ratios of RIF from ELF and AC to plasma. The final model estimated *R*_ELF/unbound-plasma_ and *R*_AC/unbound-plasma_ to be 1.28 [coefficient of variation (CV) = 10 %] and 5.5 (CV = 9 %), respectively. The model predicted the unbound concentration in ELF and AC to be higher than in plasma since the unbound ratio was predicted to be greater than 1. Interestingly, if protein binding was not accounted for, the total concentration ratios of ELF and AC in relation to plasma were 0.26 and 1.1, respectively. As such, without accounting for the protein binding, the model predicted lower ELF concentrations compared to plasma although the reverse was seen using the unbound ratio.

As reported by others, there are a number of factors that could have unwanted and in some cases undistinguishable influence over the results originating from studies using BAL [8]. The perhaps simplest example being that the efferent of the BAL method varies between studies, hence making the results to some extent incomparable [8]. Another source of error is the risk of contamination of ELF concentrations from lysis of the components that make up the ELF mixture. For example, lysis of alveolar macrophages could potentially lead to an artificially increased antibiotic concentration measured in ELF [9]. Despite the shortcomings mentioned above, the BAL method enables a relative simple way of sampling ELF from the surface of the alveolar wall and further quantification of drug concentrations in ELF and AC [8].

The possibility to sample and quantify drug concentrations close to the site of effect in the lung is of interest for pulmonary infections as the concentrations of antibiotics in ELF for extracellular pathogens and in AC, especially in macrophages, for intracellular pathogens, are thought to reflect antibiotic activity in pneumonia [9]. The concentration ratios estimated by our model describe the extent of distribution and are a good indication of the studied drug’s potential to reach the site of action and have effect at extracellular and intracellular sites in the lung. The rate of distribution to ELF and AC from plasma was in the model captured by the rate constants *k*_ELF_ and *k*_AC_. However, due to the sparseness of information in the data where only one sample was obtained in the subjects at a time point when equilibrium of distribution from plasma to the lung had occurred (4 h), the rate of distribution could not be estimated but instead an instantaneous distribution from plasma to ELF and AC was assumed. Although this study design allowed for estimation of the extent of distribution, the true rate of distribution could have been obtained using a more informative sampling design. Capturing the rate of distribution from plasma to the site of action is of great interest as it dictates if plasma is a good predictor of a PKPD relationship or not for a particular drug. If the rate of distribution is fast, the ratio of drug concentrations at the site of action and in plasma will quickly become constant. Plasma PK would in such a situation be a good marker of PK at the site of action, and it would be possible to relate concentrations in plasma (exposure) to the effect when obtaining PKPD relationships. However, in the case of a slow rate of distribution, the relationship between plasma concentrations and effect would be less relevant as the effect is governed by a concentration at the site of action which would not be directly reflected by plasma concentrations. The failure of realizing this could potentially lead to suboptimal dosing causing treatment failure and higher risk of emergence of bacterial drug resistance.

The final pharmacometric pulmonary model described RIF plasma PK including autoinduction, with the use of an enzyme turn-over model, and the distribution of RIF from plasma to ELF and AC. Our results are in agreement with previously reported results with regards to RIF’s general pharmacokinetic properties [4, 5, 26]. Typical oral clearance and volume of distribution were estimated to be 3.8 and 76.6 L, respectively. The influence of potential subpopulation-specific properties or covariates where not explored in this analysis. The effect of covariates specific to RIF and this specific population have been explored in a previous publication [6].

In summary, a pharmacometric pulmonary model was developed for quantifying the extent and rate of distribution from plasma to ELF and AC to be used for single or multiple sample designs. Quantification of the rate of distribution to ELF and AC will, in comparison to the PK in plasma, define if plasma is a good marker for drug exposure at the site of action or if PKPD relationships should be built using pulmonary PK. Information about the extent of distribution to ELF and AC will provide information about the expected drug exposure in lung. The ELF and AC submodels are not drug dependent and can as such be applied to other drugs where drug exposure in the lung is of interest. The model can also be used for further investigations of optimized BAL sampling designs.
